# Response to “Comment on ‘*In Vitro* Effects of Bisphenol A β-D-Glucuronide (BPA-G) on Adipogenesis in Human and Murine Preadipocytes’”

**DOI:** 10.1289/ehp.1510356

**Published:** 2015-12-01

**Authors:** Ella Atlas, Jonathan G. Boucher, Shaimaa Ahmed, Adèle Boudreau

**Affiliations:** Health Canada, Ottawa, Ontario, Canada

Our paper was designed to investigate the possibility that the main metabolite of bisphenol A (BPA), BPA β-D-glucuronide (BPA-G), has biological activity distinct from estrogenic activity. Our results clearly show there are effects at the cellular level in human and mouse preadipocytes starting at a concentration of 0.01 µM and peaking at 10 µM (see Figures 1 and 3).

The concentrations used in this study are often used *in vitro* for evaluation of estrogenic activity (Matthews et al. 2001), and concentrations in the range of the ones where we saw significant effects in human preadipocytes (0.05 µM; see Figure 3) were reported in human samples. The measurement 11.5 µM was a typo and should be 11.5 nM. We apologize for the error, which has been corrected in the final article.

We agree that concentrations of plasma, not urine, are to be considered. However, the methodology to accurately assess BPA-G in serum or other bodily fluids is still developing (Kosarac et al. 2012), and to measure the intracellular concentrations is beyond our laboratory’s capabilities. Nevertheless, this paper was not designed to answer the question of whether urine concentrations are correlated to plasma or serum concentrations, but rather to assess the basic question of whether BPA-G has biological activity in our *in vitro* models. The answer was yes and at nanomolar concentration in human preadipocytes. However, it is extremely difficult to correlate *in vitro* concentrations to the *in vivo* situation.

We feel, though, that Gayrard et al. have seriously misinterpreted our article and its message. We did not find, nor did we claim, that BPA-G has estrogenic activity. As a matter of fact, we could not show direct estrogenic activity in ERE-luciferase assays when the cells were treated with BPA-G. That was the case in Cos-7 cells (see Figure 4) and 3T3L1 cells (data not shown) after 48 hours of BPA-G treatment. However when the same cells, in the same experiment, were treated with free BPA, we could readily detect estrogenic activity at concentrations as low as 10 nM. If the conjugation had been reversed intracellularly, one would have expected to see some estrogenic activity when the cells were treated with BPA-G; however, we detected no such activity in any of the cells.

In addition, even if the cells and the adipose tissue could deconjugate BPA-G into free BPA, the implication is the same—that BPA-G has biological activity.
